# Hepatitis B Virus, Pneumococcal, Diphtheria, and Tetanus Vaccination Responses in Compensated Cirrhosis

**DOI:** 10.1111/liv.70644

**Published:** 2026-04-12

**Authors:** Vincent Haghnejad, Mélissa Lucas, Lucie Chevrier, Hélène Jeulin, Isabela Quilici, Sébastien Daude, Mouni Bensenane‐Oussalah, Nicolas Jacquinot, Evelyne Schvoerer, Frederic Batteux, Jean Pierre Bronowicki

**Affiliations:** ^1^ Hépato‐Gastroentérologie Centre Hospitalier Universitaire Nancy Nancy France; ^2^ INSERM U1256 NGERE Université De Lorraine Nancy France; ^3^ Plateforme D'immunomonitoring Vaccinal Service D'immunologie Biologique, Hôpital Cochin, AP‐HP Paris France; ^4^ Laboratoire De Virologie Centre Hospitalier Universitaire Nancy Nancy France

**Keywords:** cirrhosis, diphtheria, hepatitis B virus, immunity, pneumococcus, serology, tetanus, vaccination, vaccine

## Abstract

**Background and Aims:**

Cirrhosis favours infections that can lead to liver decompensation and death. Some of these infections can be prevented by vaccination. This study aimed to evaluate the immune response after HBV, pneumococcal, diphtheria, and tetanus vaccination in cirrhotic patients.

**Methods:**

Patients with cirrhosis were prescribed 3 doses of hepatitis B vaccine, a tetanus‐diphtheria booster and/or a 13‐valent pneumococcal conjugate vaccine followed by a 23‐valent polysaccharide vaccine. HBs seroconversion was assessed 6 months after the last booster dose. Antibody concentrations for seven pneumococcal serotypes, tetanus and diphtheria were measured by ELISA before (V0) and 4 to 6 months after vaccination (V1).

**Results:**

Of the 125 patients enrolled, 83 were analysed for HBs seroconversion, 119 for pneumococcal vaccine response and 117 for tetanus and diphtheria vaccine response. The anti‐HBs seroconversion rate was 31%. A doubling of the antibody was observed in 19% of cases for tetanus and in 26% of cases for diphtheria. For the pneumococcal vaccine, the global protection (at least 5 antibodies with a concentration ≥ 1.3 mg/L against the 7 serotypes of pneumococcus tested) increased from 39% before vaccination to 80% after vaccination. However, only 48% of patients had a 2‐fold increase in IgG antibody for at least five of the seven serotypes tested.

**Conclusion:**

Our study highlights a weak response to the hepatitis B, tetanus, and diphtheria vaccines and an acceptable immunological response to the pneumococcal vaccine, although lower than in healthy subjects. There is a real need to optimise vaccination in cirrhotics.

## Lay Summary

Cirrhotic patients are at very high risk of infection, which is why vaccination is recommended. Our study is the first to evaluate the response to different vaccines in these patients. It shows a very weak response to vaccination against hepatitis B, tetanus and diphtheria, and an acceptable response to the pneumococcal vaccine. There is a need to optimise vaccination schedules and to vaccinate earlier, well before the onset of cirrhosis.

**Abbreviations**
DT: diphtheria‐tetanus
HBV: hepatitis B virus
PCV13: 13‐valent pneumococcal conjugate vaccine
PPSV23: 23‐valent pneumococcal polysaccharide vaccine

## 1 Introduction

Cirrhosis is an increasing cause of morbidity and mortality in developed countries. In 2017, more than 1.32 million deaths were caused by cirrhosis worldwide [1]. Cirrhosis‐associated immune dysfunction (CAID), characterised by alterations in innate and adaptive immunity, results in an inadequate immune response to a wide range of pathogens. It is responsible for an increased risk of infections that can lead to liver decompensation and death [2]. Some of these infections can be prevented by vaccination. The Advisory Committee on Immunization Practices (ACIP) currently recommends vaccination against hepatitis A virus (HAV), hepatitis B virus (HBV), influenza, pneumococcal, herpes zoster, SARS‐CoV‐2, tetanus and diphtheria for chronic liver disease (CLD) (https://www.cdc.gov/vaccines/hcp/imz‐schedules/adult‐medical‐condition.html). However, CAID may be responsible for reduced vaccine responses. Indeed, response rates to HBV vaccination in cirrhotic patients range from 16% to 79%, compared with up to 90% in immunocompetent patients. In addition, vaccine efficacy decreases with the severity of liver disease [3]. For the hepatitis A vaccine, the seroconversion rate is approximately 95% in patients with controlled chronic liver disease, but only 71% and 57%, respectively, in those with Child‐Pugh B and C scores [4, 5]. Patients with cirrhosis who are infected with influenza virus not only have an increased risk of developing severe disease but also have a higher mortality rate compared to patients without liver disease [4, 5]. The efficacy of the influenza vaccine is good, with a seroconversion rate of 70%–80% for the A or B strain, depending on the study, and regardless of the severity of liver disease. Vaccination significantly reduces hospital admissions [6]. Invasive pneumococcal infections are more frequent and severe in patients with chronic liver disease [7]. This infection is associated with significantly higher mortality. Vaccination is therefore strongly recommended [4, 5]. In a small study, 45 patients listed for liver transplantation had a significant increase in antibodies one month after pneumococcal vaccination, but with limited efficacy over time due to a significant decrease in antibody levels at 6 months [8]. There are currently no data on the efficacy of pneumococcal vaccines in a large cohort of cirrhotic patients. In France, diphtheria and tetanus vaccines are mandatory for infants and are recommended for all adults at 25, 45 and 65 years of age and every 10 years thereafter (https://solidarites‐sante.gouv.fr/prevention‐en‐sante/preserver‐sa‐sante/vaccination/calendrier‐vaccinal). ACIP recommends a booster shot every 10 years, regardless of age. (https://www.cdc.gov/vaccines/hcp/imz‐schedules/adult‐medical‐condition.html).

Tetanus and diphtheria vaccines are highly effective in healthy adults, with protective responses of 99.6%–100% and 97.5%–98.2% after tetanus and diphtheria boosters, respectively [4]. To our knowledge, no study has evaluated the efficacy of these vaccines in cirrhotic patients.

In the Hepatology Department of the University Hospital of Nancy, cirrhotic patients are routinely vaccinated according to French recommendations. This retrospective, observational study aimed to evaluate the immune response after HBV, pneumococcal, diphtheria and tetanus vaccination in cirrhotic patients and to identify factors that may influence the response. Our aim was also to verify whether an absence of an immune response to one vaccine was associated with an absence of responses to the other three.

## 2 Patients and Methods

### 2.1 Study Population and Design

This study was a retrospective observational cohort study including consecutive cirrhotic patients eligible for hepatitis B, pneumococcal and/or diphtheria‐tetanus vaccination from September 2018 to October 2021 at the University Hospital of Nancy, France. We aimed to evaluate the immunological response after hepatitis B and pneumococcal vaccination and/or diphtheria‐tetanus (DT) booster dose in cirrhotic patients. The secondary objective was to identify factors that could predict the response to each vaccine. In particular, we aimed to establish whether a lack of response to one of the vaccines—especially the hepatitis B vaccine—was associated with a poor response to the other three vaccines.

During a routine follow‐up consultation, adult patients with cirrhosis who had never been vaccinated against hepatitis B or pneumococcus and had not recently received a diphtheria and tetanus booster, and who agreed to be vaccinated, were given a prescription for the various necessary vaccines. All hepatologists in the department used the same prescription, which was pre‐registered in the patients' electronic records.

This meant that the vaccination schedules and vaccines were the same for all patients.

The different vaccines were administered either by the general practitioner, a nurse, or the hospital vaccination centre, depending on the patient's preference. The vaccination record was checked during the next visit, six months later, to ensure that the vaccination schedule had been followed correctly.

Cirrhosis was defined by non‐invasive markers (clinical, biological, liver stiffness and/or imaging signs) or by histology after biopsy. We collected clinical and biological data available in the medical records on day 1, particularly data necessary to characterise cirrhosis. Patients on immunosuppressive treatment or with Child‐Pugh C were excluded from the analysis. Given the retrospective design of the study, French legislation mandates only the assurance of personal data protection [9]. The ‘Nancy Biochemical Database’ is registered at the French National Commission on Informatics and Liberty, CNIL, under the record No. 1763197v0, which supervises the protection of individuals with regard to the processing of personal data. The Institutional Review Board of the University Hospital of Nancy approved the study in accordance with the ethical principles of the Declaration of Helsinki.

### 2.2 Vaccines

HBV vaccine‐naive patients negative for anti‐HBs and anti‐HBc antibodies received 3 doses (20 μg/dose) of Engerix‐B (GlaxoSmithKline, France) at 0, 1, and 6 months.

Patients with no recent pneumococcal vaccination received one dose of a 13‐valent pneumococcal conjugate vaccine (PCV13; Prevenar, Pfizer, France), followed 2 months later by a 23‐valent pneumococcal polysaccharide vaccine (PPSV23; Pneumovax, MSD, France), according to French recommendations (https://solidarites‐sante.gouv.fr/prevention‐en‐sante/preserver‐sa‐sante/vaccination/calendrier‐vaccinal). The two vaccines share antigens for 12 common serotypes (1, 3, 4, 5, 6B, 7F, 9 V, 14, 18C, 19A, 19F and 23F). In addition, PCV13 covers serotype 6A and PPSV23 covers serotypes 2, 8, 9 N, 10A, 11A, 12F, 15B, 17F, 20, 22F and 33F.

The diphtheria‐tetanus (DT) vaccine was given in combination with poliovirus and pertussis as a single injection (BoostrixTetra, GlaxoSmithKline, France or Repevax, Sanofi Winthrop, France).

On the same day, patients could receive intramuscular injections of HBV vaccine and/or PCV13 and/or the tetanus‐diphtheria‐polio‐pertussis booster.

### 2.3 Immunogenicity Assessment

For the HBV vaccine, the immune response was assessed by HBs antibody titration (Atellica IM Anti‐Hepatitis B surface Antigen 2 (aHBs2), Siemens, Germany) up to 6 months after the last vaccine dose. An HBs antibody titre ≥ 10 IU/mL was considered an effective immune response.

For the other vaccines, IgG antibodies were measured just before vaccination (V0) and at the six‐monthly routine visit after the last injection for each vaccine (V1), i.e., 6 months after the DT injection and 4 months after the PPSV23 injection.

Anti‐Tetanus IgG and Anti‐Diphtheria IgG were quantified in serum samples using a commercially available kit (Sekisui Virotech, catalogue numbers EC124.00 and EC129.00, respectively). The Anti‐Tetanus toxin IgG concentrations were expressed in IU/ml following the WHO Standards. The standard curve of the Tetanus ELISA has been verified using the international standard of the WHO for human Tetanus immunoglobulin (TE‐3). The Anti‐Diphtheria toxin IgG concentrations were expressed in IU/ml following the WHO Standard. The standard curve of the Diphtheria ELISA has been verified using the Diphtheria Antitoxin Human Serum (00/496) of the Institute for Biological Standards and Control, WHO International Laboratory for Biological Standards in Great Britain.

Pneumococcal polysaccharide (PsPN)‐specific IgG antibody titers were measured by ELISA following analytical WHO‐recommendation's (www.vaccine.uab.edu), as previously described [10] for seven pneumococcal serotypes (4, 6B, 9 V, 14, 18C, 19F, and 23F) targeted by both PCV13 and PPSV23. Briefly, PsPN (SSI Diagnostica, Hillerød, Denmark) were coated in 96‐well plates (Greiner Bio‐One, Les Ulis, France). Samples, controls, and standard 007‐sp (University of Alabama, Alabama, USA) were pre‐absorbed with 5 μg/mL pneumococcal Cell Wall polysaccharide (SSI Diagnostica) and with 10 μg/mL serotype 22F capsular polysaccharide (SSI Diagnostica), then incubated for 2 h in coated plates. After washing, plates were incubated with alkaline phosphatase‐conjugated anti‐human goat IgG antibody (SouthernBiotech, Alabama, USA) for 2 h at room temperature. IgG antibodies were detected with paranitrophenyl phosphate substrate (Euromedex, Souffelweyersheim, France). The absorbance was read at 405 nm by a Multiskan FC spectrophotometer (ThermoFisher Scientific, Asnière‐sur‐Seine, France). Anti‐pneumococcus antibody titers were determined in each specimen by analysis of linear regression plots compared with the 007sp reference sample.

Immunological response to diphtheria or tetanus was defined as a twofold increase in IgG antibody titre between baseline (V0) and after vaccination (V1) (ratio V1/V0 ≥ 2). Long‐term protection was defined as an IgG antibody titre > 1 IU/mL and partial protection as an IgG antibody titre between 0.1–1 IU/mL by ELISA according to laboratory standards.

Defining seroconversion in response to pneumococcal vaccination is complex because there is no consensus on the correlates of protection. In the paediatric population, a threshold of protection of 0.35 μg/mL is commonly accepted, and this serves as a reference for all pneumococcal immunogenicity studies in children. Currently, there are no validated correlates of pneumococcal vaccine protection in adults. Several studies have defined response criteria for the pneumococcal vaccine, particularly adapted for evaluating long‐term protection in immunocompromised patients. Based on the recommendations of the American Academy of Allergy, Asthma & Immunology, an increase in antibody level of at least two‐fold was an indicator of a positive antibody response to the vaccine (i.e., the ratio of post‐ and pre‐vaccination antibody levels), and a concentration of 1.3 μg/mL was an indicator of protection [11]. Patients who developed an immunological response (a twofold increase) towards at least 70% of serotypes (at least five of the seven serotypes tested) at four months were considered responders. To evaluate long‐term protection, patients with an IgG concentration of at least 1.3 μg/mL for at least 70% of serotypes were considered protected.

### 2.4 Statistical Analysis

Geometric mean concentrations (GMCs) of serum antibodies were calculated for tetanus, diphtheria, and seven specific pneumococcal serotypes at baseline and after vaccination. The ratio of antibody concentrations between V1 and V0 was calculated for each vaccine. Qualitative variables were compared using the chi‐squared test or Fisher's exact test. Quantitative variables were compared using the Mann–Whitney U test or Student's *t*‐test. A *p*‐value less than 0.05 was considered statistically significant.

## 3 Results

### 3.1 Study Population

**FIGURE 1:**
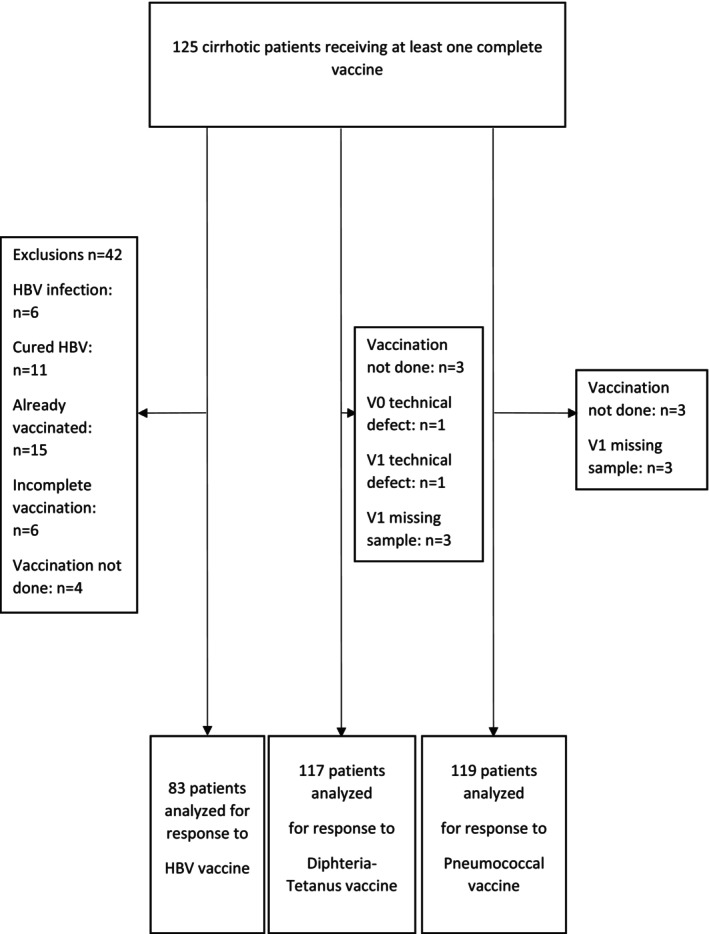
Flowchart.

A total of 125 patients who had received at least one complete vaccination were evaluated. Of these, 83 patients received the three doses of HBV vaccine and were assessed for immunological response. Immunological response to the diphtheria‐tetanus vaccine was assessed in 117 patients. For the pneumococcal vaccine, the immunological response was assessed in 119 patients (Figure 1). Among the 125 patients, 78 received the three different vaccines, 39 were vaccinated against diphtheria‐tetanus and pneumococcus, 1 against HBV and pneumococcus, 4 against HBV alone and 1 against pneumococcus alone.

The characteristics of the patients are shown in Table 1. The median age was 64 years, and 74% of patients were male. The majority of patients had Child‐Pugh A cirrhosis (84%). The median MELD score was 8. The etiologies of cirrhosis were alcohol in 55 patients, MASH in 35 patients, chronic hepatitis C in 26 cases, chronic hepatitis B in 6 cases and other causes in 8 cases. Several etiologies could be present in one patient. All patients with viral C cirrhosis had undetectable HCV RNA after direct antiviral treatment, and all patients with viral B cirrhosis had undetectable HBV DNA on nucleotide analogue.

**TABLE 1:** Characteristics of patients at baseline.

Variables	Whole population *n* = 125
Age, year, median (IQR)	64 (58; 70)
Male gender, *n* (%)	93 (74)
BMI, kg/m^2^, median (IQR)	28 (25; 33)
Child‐Pugh, *n* (%)	
Class A	105 (84)
Class B	20 (16)
MELD score, median (IQR)	8 (7; 11)
ALBI score	
Grade 1, ≤ −2.60	75 (60)
Grade 2/3, > −2.60	50 (40)
Liver stiffness, kPa, median (IQR)	20.6 (11.4; 40.7)
Fibrotest, median (IQR)	0.75 (0.64; 0.89)
Aetiology of cirrhosis, *n* (%)	
Non viral	97 (78)
Viral	28 (22)
HCC, *n* (%)	16 (13)
Diabetes, *n* (%)	58 (46)
Active heavy drinking, *n* (%)	33 (26)
Total Bilirubin, μmol/L, median (IQR)	14 (10; 22)
Albumin, g/L, median (IQR)	41 (37; 45)
Prothrombin rate, %, median (IQR)	86 (70; 94)
Blood glucose, g/L, median (IQR)	1.04 (0.87; 1.49)
HbA1c, %, median (IQR)	5.7 (5.2; 6.7)
Creatinine, mg/L, median (IQR)	7.7 (6.4; 9.0)
GFR (MDRD), mL/min/1.73 m^2^, median (IQR)	95 (80; 100)
Platelets, G/L, median (IQR)	149 (107; 198)

### 3.2 Immunological Response After Vaccination

#### 3.2.1 HBV Vaccine

Of the 83 patients who received the 3 doses of HBV vaccine, only 26 (31%) had an immunological response that was considered effective (anti‐HBs ≥ 10 IU/mL) and of these, 9 had an anti‐HBs titre ≥ 100 IU/mL. There was no significant difference between the 26 responders and the 57 non‐responders except for renal function, which was significantly better in responders (Table 2). The median blood creatinine level was 6.75 mg/L in responders and 8 mg/L in non‐responders (*p* < 0.05). Similarly, the median glomerular filtration rate estimated using the MDRD formula was 100 mL/min/1.73 m^2^ in responders and 90 mL/min/1.73 m^2^ in non‐responders (*p* < 0.05). There appeared to be no correlation between the response to the HBV vaccine and the response to the other 3 vaccines.

**TABLE 2:** Comparison of HBV vaccine responders and non‐responders.

Variable	Responders *n* = 26	Non‐responders *n* = 57	*p*
Age, years, median (IQR)	65 (61; 68)	64 (58; 70)	ns
Male gender, *n* (%)	21 (81)	43 (75)	ns
BMI, kg/m^2^, median (IQR)	31 (27; 35)	29 (26; 32)	ns
Child‐Pugh, *n* (%)			ns
Class A	22 (85)	49 (86)	
Class B	4 (15)	8 (14)	
MELD	8 (7; 10)	8 (6; 10)	ns
ALBI score			ns
Grade 1, ≤ −2.60	15 (58)	38 (67)	
Grade 2/3, > −2.60	11 (42)	19 (33)	
Liver stiffness, kPa, median (IQR)	20.5 (11.6; 39.8)	19.4 (11.4; 31.0)	ns
Fibrotest, median (IQR)	0.78 (0.64; 0.88)	0.75 (0.67; 0.89)	ns
Aetiology of cirrhosis, *n* (%)			ns
Non viral	21 (81)	46 (81)	
Viral	5 (19)	11 (19)	
HCC, *n* (%)	6 (23)	4 (7)	ns
Diabetes, *n* (%)	12 (46)	25 (44)	ns
Active heavy drinking, *n* (%)	5 (19)	15 (26)	ns
Total Bilirubin, μmol/L, median (IQR)	14 (10; 21)	14 (10; 20)	ns
Albumin, g/L, median (IQR)	41 (37; 45)	42 (38; 44)	ns
Prothrombin rate, %, median (IQR)	82 (74; 93)	89 (71; 98)	ns
Blood glucose, g/L, median (IQR)	1.06 (0.91; 1.61)	1.10 (0.87; 1.73)	ns
HbA1c, %, median (IQR)	5.7 (5.4; 7.4)	5.9 (5.5; 7.0)	ns
Creatinine, mg/L, median (IQR)	6.7 (6.2; 8.0)	8.0 (6.6; 9.7)	0.014
GFR (MDRD), mL/min/1.73 m^2^, median (IQR)	100 (90; 100.)	90 (72; 100)	0.028
Platelets, G/L, median (IQR)	138 (111; 184)	149 (112; 198)	ns
Response to tetanus vaccine, *n* (%)	5 (19)	9 (16)	ns
Response to diphtheria vaccine, *n* (%)	7 (27)	13 (23)	ns
Response to pneumococcal vaccine, *n* (%)	11 (42)	26 (46)	ns

#### 3.2.2 Tetanus Vaccine

**FIGURE 2:**
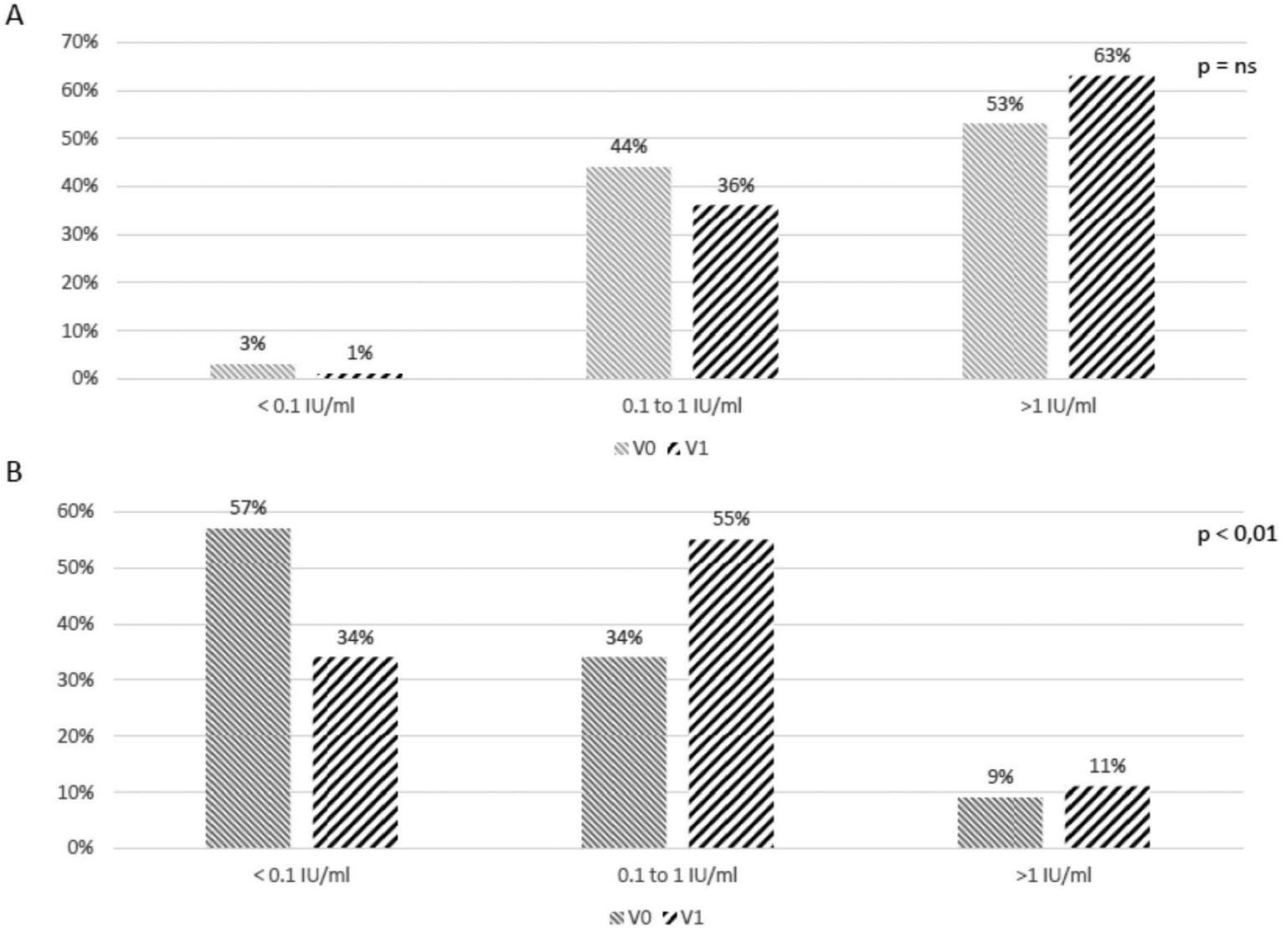
(A) Anti‐tetanus status before (V0) and after (V1) vaccination. (B) Anti‐diphtheria status before (V0) and after (V1) vaccination. IgG antibody titre > 1 IU/ml is considered as long‐term protection, and IgG antibody titre between 0.1–1 IU/ml is considered as partial protection.

At baseline (V0), 4 patients (3.4%) had an anti‐tetanus IgG concentration below 0.1 IU/mL and were considered unprotected, 51 patients (43.5%) had partial protection with an IgG concentration between 0.1 and 1, and 62 patients (53%) were considered to have long‐term protection against tetanus with an IgG concentration > 1 IU/mL. Six months after vaccination (V1), 1.7% (*n* = 2), 35% (*n* = 41), and only 64% (*n* = 75) had IgG concentrations below 0.1 IU/mL, between 0.1 and 1 IU/mL and > 1 IU/mL, respectively. The proportions were not significantly different between V0 and V1. (Figure 2A). In addition, the geometric mean (GMC) of anti‐tetanus antibodies was not significantly different between V0 and V1 (1.06 IU/mL vs. 1.32 IU/mL, ns).

Six months after tetanus vaccination, only 22 of the 117 patients (19%) had an antibody titre at least twice as high as the initial titre. These patients were considered good immunological responders. Tetanus responders were significantly more likely to respond to the pneumococcal vaccine. However, no correlation was found between tetanus response and response to HBV and diphtheria vaccines. Patients with a satisfactory immunological response had a significantly lower basal tetanus antibody concentration than non‐responders (0.44 vs. 1.30, *p* < 0.0001) (Table 3).

**TABLE 3:** Comparison of tetanus vaccine responders and non‐responders.

Variable	Responders *n* = 22	Non responders *n* = 95	*P*
Age, years, median (IQR)	65 (58; 70)	64 (57; 69)	ns
Male gender, *n* (%)	18 (82)	69 (73)	ns
BMI, kg/m^2^, median (IQR)	26 (23; 32)	29 (26; 33)	ns
Child‐Pugh, *n* (%)			ns
Class A	19 (86)	78 (82)	
Class B	3 (14)	17 (18)	
MELD	8 (7; 9)	8 (7; 11)	ns
ALBI score			ns
Grade 1, ≤ −2.60	13 (59)	56 (59)	
Grade 2/3, > −2.60	9 (41)	39 (41)	
Liver stiffness, kPa, median (IQR)	23.6 (12.6; 44.0)	18.7 (11.4; 39.4)	ns
Fibrotest, median (IQR)	0.70 (0.54; 0.84)	0.76 (0.69; 0.90)	ns
Aetiology of cirrhosis, *n* (%)			ns
Non viral	18 (82)	71 (75)	
Viral	4 (18)	24 (25)	
HCC, *n* (%)	3 (14)	11 (12)	ns
Diabetes, *n* (%)	10 (45)	43 (45)	ns
Active heavy drinking, *n* (%)	6 (27)	22 (23)	ns
Total Bilirubin, μmol/L, median (IQR)	17 (10; 23)	14 (10; 22)	ns
Albumin, g/L, median (IQR)	43 (37; 46)	41 (37; 44)	ns
Prothrombin rate, %, median (IQR)	79 (71; 93)	86 (68; 94)	ns
Blood glucose, g/L, median (IQR)	0.98 (0.85; 1.48)	1.05 (0.89; 1.48)	ns
HbA1c, %, median (IQR)	5.6 (5.3; 7.0)	5.7 (5.2; 6.6)	ns
Creatinine, mg/L, median (IQR)	7.0 (6.4; 8.3)	8.0 (6.4; 9.4)	ns
GFR (MDRD), mL/min/1.73m^2^, median (IQR)	95 (82; 100)	94 (79; 100)	ns
Platelets, G/L, median (IQR)	168 (121; 221)	140 (97; 185)	0.05
Anti‐Tetanos IgG titre V0	0.44 (0.26; 0.95)	1.30 (0.86; 2.10)	< 0.00001
Anti‐Tetanos IgG titre V1	1.90 (1.10; 2.75)	1.20 (0.82; 2.25)	ns
Response to HBV vaccine, *n* (%)	5 (36)	20 (31)	ns
Response to diphtheria vaccine, *n* (%)	7 (32)	23 (24)	ns
Response to pneumococcal vaccine, *n* (%)	15 (68)	40 (42)	< 0.05

#### 3.2.3 Diphtheria Vaccine

At baseline (V0), 63 (54%) patients were not protected with an anti‐diphtheria IgG concentration below 0.1 IU/mL, 44 (38%) patients had an anti‐diphtheria IgG concentration between 0.1–1.0 and 10 (8%) were considered to have long‐term protection with an IgG concentration > 1 IU/mL. After vaccination (V1), 34% (*n* = 40), 55% (*n* = 64) and 11% (*n* = 13) of patients had IgG concentrations less than 0.1 IU/mL, between 0.1–1.0 IU/mL and > 1 IU/mL, respectively (Figure 2B). The GMC of anti‐diphtheria antibodies increased significantly before (0.17 UI/ml) and after vaccination (0.24 UI/ml) (*p* < 0.01). Among the 107 patients who didn't have long‐term protection (IgG concentration ≤ 1 mg/L) before vaccination, only 8 (7%) had long‐term protection after vaccination. Only 30 of the 117 patients (26%) had an antibody titre at least twice as high as the initialtitre. There were no significant differences between responders and non‐responders. Diphtheria responders were significantly more likely to respond to the pneumococcal vaccine. However, no correlation was found between response to the diphtheria vaccine and response to HBV and tetanus vaccines (Table 4).

**TABLE 4:** Comparison of diphtheria vaccine responders and non‐responders.

Variable	Responders *n* = 30	Non‐responders *n* = 87	*P*
Age, years, median (IQR)	62 (56; 68)	65 (58; 70)	ns
Male gender, *n* (%)	25 (83.3%)	62 (71.3%)	ns
BMI, kg/m^2^, median (IQR)	28 (25; 32)	28 (26; 33)	ns
Child‐Pugh, *n* (%)			ns
Class A	25 (83)	72 (82)	
Class B	5 (17)	15 (17)	
MELD	8 (7; 10)	8 (7; 11)	ns
ALBI score			ns
Grade 1, ≤ −2.60	19 (63)	50 (57)	
Grade 2/3, > −2.60	11 (37)	37 (43)	
Liver stiffness, kPa, median (IQR)	32.4 (10.1; 44.0)	19.0 (11.4; 38.0)	ns
Fibrotest, median (IQR)	0.77 (0.63; 0.90)	0.75 (0.66; 0.89)	ns
Aetiology of cirrhosis, *n* (%)			ns
Non viral	20 (67)	69 (79)	
Viral	10 (33)	18 (21)	
HCC, *n* (%)	2 (7)	12 (14)	ns
Diabetes, *n* (%)	14 (47)	39 (45)	ns
Active heavy drinking, *n* (%)	4 (13)	24 (28)	ns
Total Bilirubin, μmol/L, median (IQR)	13 (10; 22)	14 (10; 22)	ns
Albumin, g/L, median (IQR)	42 (36; 46)	41 (37; 44)	ns
Prothrombin rate, %, median (IQR)	82 (70; 93)	86 (70; 98)	ns
Blood glucose, g/L, median (IQR)	0.99 (0.90; 1.60)	1.05 (0.86; 1.47)	ns
HbA1c, %, median (IQR)	5.5 (5.2; 7.1)	5.7 (5.3; 6.6)	ns
Creatinine, mg/L, median (IQR)	8.0 (6.5; 8.8)	7.7 (6.4; 9.5)	ns
GFR (MDRD), mL/min/1.73m^2^, median (IQR)	90 (84; 100)	95 (79; 100)	ns
Platelets, G/L, median (IQR)	168 (112; 187)	144 (100; 195)	ns
Anti‐diphtheria IgG titre V0	0.13 (0.09; 0.29)	0.09 (0.09; 0.29)	ns
Anti‐ diphtheria IgG titre V1	0.57 (0.35; 1.58)	0.10 (0.09; 0.28)	< 0.00001
Response to HBV vaccine, *n* (%)	7 (23)	15 (17)	ns
Response to tetanos vaccine, *n* (%)	7 (35)	18 (31)	ns
Response to pneumococcal vaccine, *n* (%)	24 (80)	31 (36)	< 0.00001

#### 3.2.4 Pneumococcal Vaccine

**FIGURE 3:**
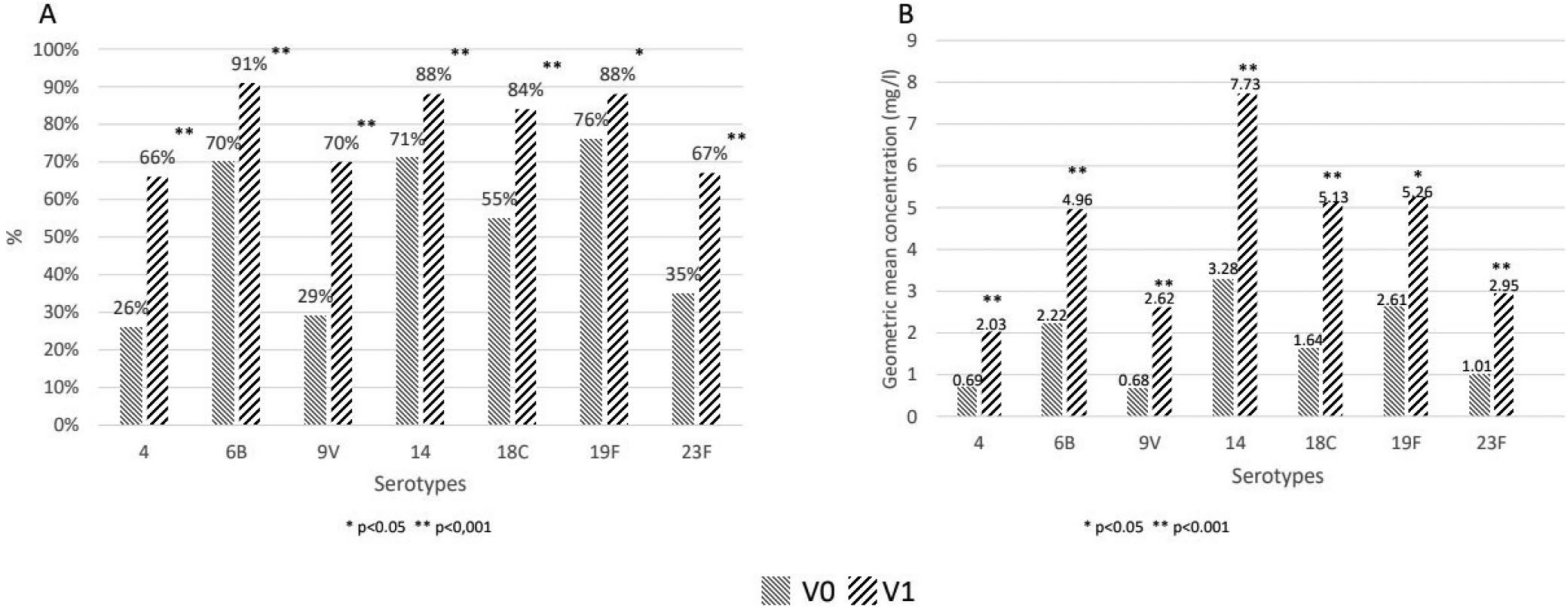
(A) Proportion of patients with antibodies ≥ 1.3 μg/ml before (V0) and after (V1) vaccination according to the different serotypes. (B) Geometric means of IgG titers against the different serotypes before (V0) and after (V1) vaccination.

Despite no known vaccination against pneumococcus, 47 (39%) patients had at least 5 antibodies with a concentration ≥ 1.3 μg/mL against the 7 serotypes of pneumococcus tested at baseline. Following vaccination, the number of patients considered to have global protection increased to 95 (80%). Figure 3 shows the proportion of antibodies with a concentration ≥ 1.3 μg/mL (Figure 3A) and the geometric means at V0 and V1 (Figure 3B) for each specific serotype. Only 57 of the 119 patients (48%) had a 2‐fold increase in IgG antibody (V1/V0 ≥ 2) for at least five of the seven serotypes tested. There were no significant differences between responders and non‐responders. Responders were significantly more likely to respond to tetanus and diphtheria. No correlation was found between response to the pneumococcal vaccine and response to the HBV vaccine (Table 5).

**TABLE 5:** Comparison of pneumococcal vaccine responders and non‐responders.

Variable	Responders *n* = 58	Non‐responders *n* = 61	*P*
Age, years, median (IQR)	64 (56; 70)	64 (58; 69)	ns
Male gender, *n* (%)	41 (72)	47 (76)	ns
BMI, kg/m^2^, median (IQR)	28 (25; 32)	28 (25; 33)	ns
Child‐Pugh, *n* (%)		50 (81)	ns
Class A	49 (86)	12 (19)	
Class B	8 (14)		
MELD	8 (7; 10)	8 (7; 12)	ns
ALBI score	38 (67)	32 (52)	ns
Grade 1, ≤ −2.60	19 (33)	30 (48)	
Grade 2/3, > −2.60			
Liver stiffness, kPa, median (IQR)	23.0 (9.0; 39.8)	19.6 (12.4; 46.9)	ns
Fibrotest, median (IQR)	0.75 (0.63; 0.86)	0.78 (0.68; 0.91)	ns
Aetiology of cirrhosis, *n* (%)	40 (70)	51 (82)	ns
Non viral	17 (30)	11 (18)	
Viral			
HCC, *n* (%)	4 (7.0)	10 (16)	ns
Diabetes, *n* (%)	24 (42)	30 (48)	ns
Active heavy drinking, *n* (%)	13 (23)	17 (27)	ns
Total Bilirubin, μmol/L, median (IQR)	14 (10; 23)	14 (9; 21)	ns
Albumin, g/L, median (IQR)	42 (39; 45)	40 (36; 44)	ns
Prothrombin rate, %, median (IQR)	84 (70; 92)	86 (69; 98)	ns
Blood glucose, g/L, median (IQR)	0.97 (0.86; 1.33)	1.09 (0.93; 1.59)	ns
HbA1c, %, median (IQR)	5.6 (5.2; 6.5)	5.7 (5.4; 7.0)	ns
Creatinine, mg/L, median (IQR)	7.3 (6.0; 8.9)	8.2 (6.5; 9.6)	ns
GFR (MDRD), mL/min/1.73m^2^, median (IQR)	97 (79; 100)	91 (79; 100)	ns
Platelets, G/L, median (IQR)	148 (110; 186)	147 (100; 193)	ns
Response to HBV vaccine, *n* (%)	11 (28)	14 (35)	ns
Response to tetanos vaccine, *n* (%)	15 (27)	7 (11)	0.034
Response to diphtheria vaccine, *n* (%)	24 (44)	6 (10)	< 0.00001

## 4 Discussion

In this study, we assessed the vaccine response to HBV, diphtheria, tetanus and/or pneumococcus in 125 cirrhotic patients. It is the first study to evaluate the immunological response to different vaccines in cirrhosis.

Despite a population predominantly classified as Child‐Pugh A (84%) and the exclusion of Child‐Pugh C, the response rate to HBV vaccination in our study was only 31%. In a pooled analysis of 11 studies [3, 12, 13, 14, 15, 16, 17, 18, 19, 20, 21, 22] involving 961 cirrhotic patients vaccinated with different HBV vaccine regimens and doses, serological response rates ranged from 16% to 87%. In patients who received the standard dose of vaccine (20 μg vaccine/dose), seroprotection rates ranged from 16% to 79% (mean response rate 38%), and those who received a double dose achieved relatively better seroprotection rates (range: 26%–87%; mean response rate 53%). The distribution of Child‐Pugh scores was described in 8 studies with a total of 778 patients [12, 14, 16, 17, 19, 20, 21, 22]. Of these, 30% were Child‐Pugh C, 46% were Child‐Pugh B, and only 24% were Child‐Pugh A. Seroprotection rates ranged from 26% to 70%. In addition, the mean age in these studies ranged from 41.6 to 53.6 years, compared with 64 years in our study [3]. A meta‐analysis has shown that older age is a predictor of a poor response to the HBV vaccine [23].

We conducted a study comparing the response after three standard doses of Engerix (20 μg at M0, M1, and M6) in 96 patients with inflammatory bowel disease (IBD) and 68 healthy controls. The anti‐HBs antibody rate > 10 IU/mL was 80.2% in IBD patients and 94.1% in healthy volunteers (*p* = 0.0115). In this study, immunosuppressive treatments did not appear to influence the response to the vaccine [24]. Table S1 summarises the results of various studies that evaluated the immunogenicity of different recombinant hepatitis B virus (HBV) vaccines when administered intramuscularly to patients with cirrhosis and to different populations with comorbidities.

In France, there is no recommendation for the use of an extended vaccination schedule in cirrhotic patients, as in the case for patients with renal failure, who are offered a double injection of 20 μg of vaccine antigen by IM at M0, M1, M2 and M6 [25]. Therefore, in our daily practice, we use a standard schedule of one 20 μg antigen IM injection at M0, M1 and M6, as recommended by the French health authorities. In addition to the use of double doses [26], several strategies have been explored to improve the response rates to hepatitis B vaccines, including administration of additional doses or intradermal vaccination [27]. Several studies have shown that intradermal hepatitis B vaccination can enhance immune responses in people living with HIV [26] and in patients undergoing dialysis [28]. Only one study evaluated an intradermal vaccine with a high dose (40 μg) in patients with chronic liver disease who had not responded to standard vaccination. Seroconversion was observed in 29/42 (69%) patients [29]. However, intradermal injections require specialised training and are rarely used in clinical practice. In addition, the advantage of intradermal injections over intramuscular injections seems to diminish after long‐term follow‐up [28, 30]. Several new hepatitis B vaccines have been developed in recent years that contain more potent adjuvants or additional HBsAg to increase immunogenicity [27]. One of these new vaccines is a vaccine with a cytosine phosphoguanine adjuvant (HepB‐CpG vaccine uses cytidine phosphate guanosine oligodeoxynucleotide 1018, which is a toll‐like receptor 9 agonist). Several Phase 3 studies have shown that HepB‐CpG significantly increased seroprotection rates compared to Engerix‐B in healthy volunteers [31], HIV‐positive patients [32] and patients with chronic kidney disease [33]. To our knowledge, no study has been conducted in cirrhotic patients. This vaccine is not covered by the French healthcare system.

Our study is the first to evaluate the response to diphtheria and tetanus vaccination in cirrhotic patients. We have shown that these patients have a very poor response to these vaccines. Six months after tetanus vaccination, only 19% of patients had an antibody titre at least twice as high as the initial titre. The proportion of patients with high antibody titre (> 1 IU/mL) rose from 53% before vaccination to 64% after vaccination. This seems significantly lower than the response observed in healthy subjects. A doubling of the antibody titre was observed in 91% of healthy volunteers after vaccination, and the proportion of subjects with long‐term protection rose from 57.5% to 99.5% [34]. A multicentre study compared the immunological response to the tetanus vaccine in patients with inflammatory rheumatic diseases and healthy subjects [35]. The basal rate of long‐term protection in patients was 58%, significantly lower than in healthy subjects (68%). At 3 months after vaccination, this rate increased to 87% in patients and 96% in healthy volunteers (*p* < 0.001). One month after vaccination, the proportion of subjects with at least a doubling of their antibody titre was 76% in healthy subjects. Similar percentages were found in untreated or corticoid‐treated patients. In contrast, this rate was only 18% in patients treated with rituximab and 54% in patients treated with methotrexate [35].

We also observed a very low response to the diphtheria vaccine. The proportion of subjects with high antibody titre (> 1 IU/mL) rose from 8% to 11%, and only 26% had an antibody titre at least twice as high as the initial titre after vaccination. In healthy volunteers, the proportion of responders has been reported to almost double after vaccination, with rates of around 90% [36]. In the study comparing healthy volunteers with patients with inflammatory rheumatological diseases, the rate of long‐term protection at baseline was 5% in both populations. This rate increased to 25% in both groups 3 months after vaccination. One month after vaccination, the proportion of subjects with at least a doubling of their antibody titre was 72% in healthy subjects [35]. Similar percentages were found in untreated or corticoid‐treated patients. In contrast, this rate was significantly lower in patients treated with rituximab or methotrexate [35]. Tables S2 and S3 summarise the results of various studies that evaluated the immunogenicity of tetanus and diphtheria vaccines in populations affected by comorbidities.

To our knowledge, our study is only the third to investigate the response to pneumococcal vaccination in cirrhotic patients and the first to evaluate the immunological response after sequential vaccination with PCV‐13 and PPSV‐23 vaccines. This sequential vaccination schedule was recommended by the French health authorities for older patients or those with comorbidities. This schedule was also recommended by the ACIP in the United States in 2014 for patients aged ≥ 65 years. Nearly half of the patients had global protection at baseline. After vaccination, this proportion increased to 82%. Nevertheless, only 48% had a 2‐fold increase in IgG antibody for at least five of the seven serotypes tested. Because of the different vaccine regimens and/or techniques used to assess the immune response, it is difficult to strictly compare our results with those obtained in cirrhosis and other populations. The vaccine regimen we used is probably the most effective in eliciting the strongest immune response in immunocompromised patients. In pneumococcal vaccine–naïve adults 60–64 years of age evaluated by opsonophagocytosis assay, an initial PCV13 augmented the antipneumococcal response to subsequent administration of PPSV23 for many of the serotypes in common to both vaccines. In contrast, an initial PPSV‐23 resulted in a diminished response to subsequent administration of PCV13 for all serotypes [37]. In cirrhosis, a first study has compared 15 patients with alcohol‐related cirrhosis, 10 patients with chronic obstructive pulmonary disease and 10 healthy subjects. All received a single dose of PPSV 14‐valent vaccine. At 4 and 12 weeks after vaccination, antibody titers were similar in the 3 groups. The authors concluded that patients with alcoholic cirrhosis had adequate antibody production [38]. McCashland et al. compared 45 cirrhotic patients being evaluated for liver transplantation with 13 controls [8]. All received one dose of the PPSV‐23 vaccine. After a significant increase in antibody titre one month after vaccination, a significant decrease was observed after 6 months in the cirrhotic patients. In the 25 patients evaluated after transplantation, the antibody titre was either similar to or lower than the pre‐vaccination basal level. The authors questioned the efficacy of this vaccination, especially in cases of liver transplantation [8]. Although conjugate vaccines are more effective than polysaccharide vaccines [39], no study has evaluated the efficacy of these vaccines in cirrhotic patients. Antibody responses to PCV13 in individuals with immunocompromising and high‐risk conditions are variable and generally lower compared with those of healthy controls; nevertheless, the vaccine is immunogenic. The overall protection rate in well‐controlled HIV‐infected patients who received one dose of PCV13 was 72%, six months after vaccination [10]. Half of this population was considered to be globally protected at baseline. In 2021, ACIP recommended the use of a single dose of 20‐valent PCV for PCV‐naive adults who are either aged ≥ 65 years or aged 19–64 years with certain underlying conditions [39]. This vaccine has been reimbursed in Europe since 2024. No data are currently available for this single‐dose vaccine schedule in cirrhotic patients. Table S4 summarises the key published studies that have evaluated the immunogenicity of sequential vaccination with PCV13, followed by PPSV23, in different adult populations who are at high risk of pneumococcal infection.

The second aim of this study was to look for factors predictive of immunological non‐response for each vaccine. No common risk factors of non‐response were found for the different vaccines. The only independent factor for poorer response to HBV vaccine was a moderate reduction in renal function. Severe renal insufficiency is a known factor for non‐response to the HBV vaccine [40]. Although the glomerular filtration rate was significantly lower in non‐responders, none of the patients in our study had severe renal impairment. Probably because of the high age of our population and the presence of severe chronic liver disease, there was no difference between responders and non‐responders with respect to known non‐response factors such as age, male sex, obesity, or comorbidities such as diabetes or excessive active alcohol consumption [23, 41, 42]. We did not find any correlation between the response to the HBV vaccine and the response to the other 3 vaccines.

Contrary to previous publications, age was not a factor in non‐response to the tetanus vaccine in our study. On the other hand, we have confirmed that a high basal titre of anti‐tetanus antibodies is a factor for a lower immune response [34].

For the diphtheria and pneumococcal vaccines, there was no difference in patient characteristics between immunological responders and non‐responders.

There was an association between pneumococcal vaccine response and tetanus and diphtheria vaccine responses.

The main limitation of our study is that the immunological response was only assessed by ELISA antibody titration before and after vaccination. We did not use functional tests such as opsonophagocytosis assays for pneumococcus, and we have no way of comparing the immunological response with the residual risk of infection after vaccination. Nevertheless, it gives an idea of the immunogenicity of different vaccines in relatively well‐compensated cirrhosis. It is well established that immune markers can be used as substitute endpoints for vaccine‐induced protection in immunocompetent subjects (World Health Organization. Correlates of vaccine‐induced protection: Methods and implications. Geneva: WHO; 2013. https://iris.who.int/bitstream/handle/10665/84288/WHO_IVB_13.01_eng.pdf?sequence=1).

All Phase 3 studies testing different vaccines initially focus solely on evaluating their immunogenicity. The efficacy of these vaccines in terms of prevention was subsequently confirmed by case–control studies, which validated the relevance of these strategies. However, to date, no real study has evaluated the protective efficacy of these vaccines in cirrhotic patients who have shown a satisfactory immunological response.

Our study confirms the weak response to the HBV vaccine in cirrhotic patients. It highlights an acceptable immunological response to the pneumococcal vaccine, although lower than in healthy subjects, and a weak response to the tetanus and diphtheria vaccines. These results argue in favour of more stringent vaccination schedules to be defined and, in particular, the need to offer these vaccines to patients with chronic liver disease as early as possible, well before the onset of cirrhosis.

## Author Contributions

Concept and design (JPB), acquisition, analysis and interpretation of data (VH, ML, LC, HJ, IQ, NJ, ES, FB, JPB), drafting the article (VH, ML, JPB), revising and approving the article (VH, ML, LC, HJ, IQ, SD, MBO, NJ, ES, FB, JPB).

## Funding

The authors have nothing to report.

## Conflicts of Interest

The authors declare no conflicts of interest.

## Supporting information


**Table S1:** Summary of key published studies evaluating the immunogenicity of intramuscular recombinant HBV vaccine in different adult populations.
**Table S2:** Summary of key published studies evaluating the immunogenicity of tetanus vaccine in different adult populations.
**Table S3:** Summary of key published studies evaluating the immunogenicity diphtheria vaccine in different adult populations.
**Table S4:** Summary of key published studies evaluating the immunogenicity of sequential vaccination with PCV13 followed by PPSV23 in different adult populations at high risk of pneumococcal infection.

## Data Availability

The data that support the findings of this study are available from the corresponding author upon reasonable request.
