# Supplementary material for: Hepatitis B Virus, Pneumococcal, Diphtheria, and Tetanus Vaccination Responses in Compensated Cirrhosis
Source: Liver Int. 2026 Apr 12;46(5):e70644. doi: 10.1111/liv.70644 (PMC13071121; doi:10.1111/liv.70644)
Supplement: Supplementary file 1 — Table S1: Summary of key published studies evaluating the immunogenicity of intramuscular recombinant HBV vaccine in different adult populations. Table S2: Summary of key published studies evaluating the immunogenicity of tetanus vaccine in different adult populations. Table S3: Summary of key published studies evaluating the immunogenicity diphtheria vaccine in different adult populations. Table S4: Summary of key published studies evaluating the immunogenicity of sequential vaccination with PCV13 followed by PPSV23 in different adult populations at high risk of pneumococcal infection. [file LIV-46-0-s001.docx]

**Supplementary table 1 : Summary of key published studies evaluating the immunogenicity intra-muscular recombinant HBV vaccine in different adult populations**

| **Cirrhosis** | | | | | | | | | |
| --- | --- | --- | --- | --- | --- | --- | --- | --- | --- |
| Study | Design | Patients | Age, yr | Child-Pugh A/B/C | Vaccine | Schedule | Response after complete vaccine | Response according CP | Multivariate analysis |
| Present study | Retrospective | 119 | 64 (58-70) | 71/12/0 | Engerix® | 20µg x 3 (M0,M1,M2) | 31 % | CPA 31%  CPB 33% | - |
| Chalasani N^1^ | Retrospective | 57 | 41.6±14.3 | NR | Engerix® | 20µg x 3 (M0,M1,M2) | 16% | - | - |
| Villeneuve E^2^ | Retrospective | 49 | 49±2 | 0/16/33 | Engerix® | 20µg x 3 (M0,M1,M2) | 28% |  | No factor |
| Roni DA^3^ | Prospective | 52 | 47.5±9.5 | 25/27/0 | Shanvac-B® | 20µg x 3 (M0,M1,M2) | 79% | CPA 92%  CPB 67% | - |
| Kallinowski B^4^ | Prospective | 20 | 46±12 | 7/13/- | Recombinant | 20µg x 3 (D0,D7,D21) | 36% |  |  |
| Engler SH^5^ | Prospective | G1: 14  G2: 20 | 49±10  50±6 | 0/8/6  7/6/7 | Engerix® | 20µg x 3 (D0,D7,D21)  20µg x 3 (D0,D7,D21) | 31%  26% | - | - |
| Dominguez M^6^ | Prospective | 62 | 52±9 | CP≥B8 | Engerix® | 40µg x 3 (M0,M1,M2) | 44% | - | - |
| Macedo G^7^ | Prospective | 24 | NR | NR | Engerix® | 40µg x 3 (M0,M1,M2) | 87% | - | - |
| Rodrigues-Tajes S^8^ | Prospective | 196 | 60 (53-66) | 48/105/43 | Hbvaxpro® | 40µg x 3 (M0,M1,M2) | 23% | - | No factor |
| Bonazzi PR^9^ | Retrospective | 43 | 44.2 | 8/25/10 | Recombinant | 40µg x 3 (M0,M1,M6) | 67% | CPA 37.5%  CPB 68%  CPC 90% | No factor |
| Horlander JC^10^ | Retrospective | 140 | 48.1±9.1 | 19/61/20 | Engerix® | 40µg x 4 (M0,M1,M2,M6) | 37% | No link with CP | - |
| Pascasio JM^11^ | Retrospective | 202 | 52.8±9.1 | 38/93/71 | Engerix® | 40µg x 4 (M0,M1,M2,M6) | 31.5% |  | No factor |
| Gutierrez Domingo I^12^ | Retrospective | 278 | 53,6±9.3 | 92/114/72 | Engerix® or  Recombivax® | 40µg x 4 (M0,M1,M2,M6) | 41% | CPA 53.8%  CPB 33.3%  CPC 30.1% | Diabetes  MELD |
| Arslan M^13^ | Prospective | 356 | 48.9±0.7 | 86/192/72 | Engerix® | 40µg x 4 (W0,W2,M1,M6) | 38.7% |  | - |
| **Chronic Liver Diseases** | | | | | | | | | |
| Study | Design | Patients | Age, yr | Subpopulation of interest | Vaccine | Schedule | Response after complete vaccine | Response in subpopulation | Multivariate analysis |
| De Maria N^14^ | Prospective | 224 CLD | 50.3-53.2 | 138 no cirrhosis  86 cirrhosis | Engerix® | 40µg x 3 (M0,M1,M2) | 62%  74%  34% | HCV+ 72%  Non viral 40% | - |
| Leroy V^15^ | Prospective | 77 HCV+ vs 231 HS | 42.3±8.8  42.0±8.5 | 0 cirrhosis | Engerix® | 20µg x 3 (M0,M1,M2) | 63.6%  93.9% |  | HCV infection,  Age, weight |
| Wiedman M^16^ | Prospective | 59 HCV+  vs 58 HS | 42 (31-70) | 7 cirrhosis | GenH-B-Vax® | 10µg x 3 (M0,M1,M6) | 69%  91% | Cirrhosis 16.7% (3/7) |  |
| Arbizu EA^17^ | Prospective | 38 HCV+ | 52.5±13.6 | 0 cirrhosis | Engerix® | 20µg x 3 (D0,D15,M1) | 72% |  |  |
| Horta D^18^ | Prospective | 125 CLD | 61.8±9.4 | 71 no cirrhosis  54 cirrhosis | Hbvaxpro® or Fendrix® | 40µg x 3 (M0,M1,M2)  20µg x 4 (M0,M1,M2,M6) | 76.8%  93%  55.5% | CPA 56% (23/41)  CPB 54% (7/13) | Cirrhosis |
| Medeiros RP^19^ | Prospective | G1 64 HCV+  G2 77 HCV+  G3 70 HS | 52 (39-61)  52 (41-57)  45 (38-56) | 0 cirrhosis | Butang® | 40µg x 3 (M0,M1,M6)  20µg x 3 (M0,M1,M6)  20µg x 3 (M0,M1,M6) | 76.7%  73.5%  91.2% |  | Age  Caucasian  Genotype 1 |
| Amjad W^20^ | Retrospective | G1 106  G2 60 | 59±11.3 | 34% cirrhosis | Engerix®  Heplisav® | 20µg x 3 (M0,M1,M6)  20µg x2 (M0,M1) | 45%  63% |  | Cirrhosis  COPD  Renal failure  Heplisav |
| Kwon CS^21^ | Retrospective | 120 | 58±11.1 | 86.7% cirrhosis | Heplisav® | 20µg x2 (M0,M1) | 67.5% |  | Age>50/gender  diabetes/cirrhosis |
| HIV+ patients | | | | | | | | | |
| Study | Design | Patients | Age, yr | Subpopulation of interest | Vaccine | Schedule | Response after complete vaccine | Response in subpopulation | Multivariate analysis |
| Cruciani M^22^ | Prospective | 65 | 41±8 |  | Hbvaxpro® | 40µg x 3 (M0,M1,M2) | 60% |  | Gender; CD4  Viral load |
| Launay O^23^ | Prospective | G1 145  G2 148 | 43 (19-74)  42 (19-65) | 14.7% CD4≤350  77.8% HIV-RNA< 50 c/ml | Engerix® | 20µg x 3 (M0,M1,M6)  40µg x 4 (M0,M1,M2,M6) | 65%  82% |  | Sex/ Age  Vaccine dose  Smoking/CD4  HIV RNA |
| Chaiklang K^24^ | Prospective | G1 44  G2 44  G3 44 | 41.0±6,3  42.2±7.6  41.0±6.2 | 100% CD4>200  100% HIV-RNA- | Hepavax-Gene® | 20µg x 3 (M0,M1,M6)  20µg x 4 (M0,M1,M2,M6)  40µg x 4 (M0,M1,M2,M6) | 88.6%  93.2%  95.4% |  | - |
| Reich Medeiros AF^25^ | Retrospective | 201 | 43.9±12.7 | CD4+ 467±141  HIV-RNA 24,028 | Euvax® | 20µg x 4 (M0,M1,M2,M6) | 80% |  | No factor |
| **Chronic kidney diseases** | | | | | | | | | |
| Study | Design | Patients | Age, yr | Subpopulation of interest | Vaccine | Schedule | Response after complete vaccine | Response in subpopulation | Multivariate analysis |
| Fleming SJ^26^ | Prospective | 83 | 65 (19-77) | 18% PD / 82% HD | Engerix® | 20µg x 3 (M0,M1,M6) | 27.7% |  |  |
| Bel’eed K^27^ | Retrospective | 227 | 59±16.7% | 24%PD/ 60% HD | Engerix® | 40µg x 4 (M0,M1,M2,M3) | 64.3% | HD 66.2%  PD 65.4% |  |
| Janssen JM^28^ | Prospective | 260  247 | 61.3±9.7  61.4±9 | 13.8% HD  67.4% diabetes | Engerix®  Heplisav® | 40µg x 4 (M0,M1,M2,M6)  20µg x3 (M0,M1,M6) | 81.8%  89.9% |  |  |
| **Diabetes** | | | | | | | | | |
| Study | Design | Patients | Age, yr | Subpopulation of interest | Vaccine | Schedule | Response after complete vaccine | Response in subpopulation | Multivariate analysis |
| Jackson S^29^ | Prospective | Diabetes 381/2782  763/5592 | 54±11.7 | 25.2% age≥60  47.9% BMI≥30 | Engerix®  Heplisav® | 20µg x 3 (M0,M1,M6)  20µg x2 (M0,M1) | Diabetes + vs -  65.1% vs 83.9%  90% vs 96.2% |  |  |
| Van Der Meeren O^30^ | Prospective | 378 vs  189 HS | 53.7±12.1  53.1±12.9 | 19.6% age≥60  43.3% BMI≥30 | Engerix® | 20µg x 3 (M0,M1,M2) | 75%  82% |  | BMI  Age |
| Han B^31^ | Prospective | G1 106  G2 116  G3 70 HS | NR | 34% age≥60  35.3% age≥60  20% age≥60 | D20SC  D20CHO  D20SC | 20µg x 3 (M0,M1,M6)  20µg x 3 (M0,M1,M6)  20µg x 3 (M0,M1,M6) | 89.6%  91.4%  97.1% |  | No factor |
| **Inflammatory bowel diseases** | | | | | | | | | |
| Study | Design | Patients | Age, yr | Subpopulation of interest | Vaccine | Schedule | Response after complete vaccine | Response in subpopulation | Multivariate analysis |
| Belle A^32^ | Prospective | 96 vs  68 HS | 44.9±13.4  31.0±8.3 |  | Engerix® | 20µg x 3 (M0,M1,M6) | 80.2%  94.1% |  |  |
| Singh AK^33^ | Prospective | G1 44  G2 41 | 36.9±12  35.2±11.8 | 89.8% UC  28,4% no immunosuppression | Engerix®  Engerix® | 20µg x 3 (M0,M1,M6)  40µg x 3 (M0,M1,M6) | 57%  93% |  | Dose  Anti-TNF |
| Etzion O^34^ | Prospective | G1 37  G2 35 | 38±12.7  37.6±15.5 | 79.1% CD  11.1% anti-TNF | Engerix®  Sci-B-vac® | 20µg x 3 (M0,M1,M6)  10µg x 3 (M0,M1,M6) | 81.1%  68.6% |  | Anti-TNF |
| **Healthy subjects with Age > 40 years** | | | | | | | | | |
| Study | Design | Patients | Age, yr | Subpopulation of interest | Vaccine | Schedule | Response after complete vaccine | Response in subpopulation | Multivariate analysis |
| Heyward WL^35^ | Prospective | Age 40-70  G1 451  G2 1818 | 54 | 39.8% age 50-59  27.7% age 60-70 | Engerix®  Heplisav® | 20µg x 3 (M0,M1,M6)  20µg x2 (M0,M1) | 72.8%  94.8% |  | - |
| Sablan BP^36^ | Prospective  Asian population | Age 40-70  G1 206  G2 206 | 49.1±7  49.9±7.4 |  | Engerix®  Heplisav® | 20µg x 3 (M0,M1,M6)  20µg x3 (M0,M2,M6) | 73.1%  100% | Age > 55 yr  56%  100% | - |
| Vesikari T^37^ | Prospective | G1 811  G2 796 | 56.6 (18 -90) | 36.6% BMI> 30 | Engerix®  Sci-B-vac® | 20µg x 3 (M0,M1,M6)  10µg x 3 (M0,M1,M6) | 76.5%  91.4% | Age ≥65 yr  64.7%  83.6% | - |

**Supplementary table 2 : Summary of key published studies evaluating the immunogenicity tetanus vaccine in different adult populations**

| Study | Population | Age | Vaccine | Timepoint  After vaccine | Pre-vaccine Ab concentration | | Post-vaccine Ab concentration | | Booster  response (definition) | Booster  response (%) |
| --- | --- | --- | --- | --- | --- | --- | --- | --- | --- | --- |
|  |  |  |  |  | ≥ 0.1 IU/mL | ≥1  IU/mL | ≥ 0.1 IU/mL | ≥1 IU/mL |  |  |
| Present study | 119 cirrhosis | 64 (58-70) | Tdap | 6 months | 96.6% | 53% | 98.3% | 64% | V1/V0≥2 | 19% |
| **Healthy adults** | | | | | | | | | | |
| Halperin SA | 746 healthy adults | 33 (20-72) | Tdap | 1 month | 95% | 47% | 99.9% | 98.6% | V1/V0≥2 or V1/V0≥4 | 82% |
| Thierry-Carstensen B^38^ | 800 healthy adults | 26.1 (18-55) | 401Tdap  399 Td | 1 month | 92.1% | NR | 100% | NR | V1/V0≥2 or V1/V0≥4 | 91.2% |
| Van Damme P^39^ | 293 Adults ≥ 55 yr | 64.5±7.3 | Boostrix® | 1 month | 53.4% | NR | 94.5% | NR | NR | NR |
| Weston WM^40^ | 887 adults ≥ 65 yr | 71.6±5.3 | Tdap | 1 month | 80.8% | 48.6% | 96.8% | 88.8% | NR | NR |
| **Patients with comorbidities** | | | | | | | | | | |
| Buhler S^41^ | 284 rheumatic diseases  253 healthy control | 52±14.4  45±15 | Td | 3 months | 87%  94%* | 58%  68%* | 98%  100% | 87%  96%* | V1/V0 ≥2 at 1 month | 73% |
| Nunes MC^42^ | 91 pregnant HIV+  136 pregnant HIV- | 27.3±5.5  30.7±4.9 | Adacel® |  | 100%  99.3% | 85.7%  94.9%* | 100%  100% | 100%  100% | NR | NR |
| Conrad A^43^ | 106 Allo-HSCT | 47 (37.8-60) | DTaP(±HB) -IPV-Hib# | 3.5 months | 75% | NR | 98% | NR | NR | NR |
| Epaulard O^44^ | 104 Allo-HSCT | 58 (48-64) | DTaP(±HB) -IPV-Hib# | 1 month | 100% | NR | 97.2% | NR | NR | NR |
| Guerin A^45^ | 66 patients under hemodialysis | NR | T at  M0,M1,M6 | After last boost  6 months after last boost | 39.4%§ | NR | 96.5%  62%§ | NR | NR | NR |
| Girndt M^46^ | 23 CKD  27 dialysis  7 renal transplant  15 healthy controls | 59.5±12.6  56.5±11.6  47.8±8.4  51.3±13.3 | T at  M0,M1,M6 | 1 month | NR | NR | 55%  69%  85%  100%$ | NR | NR | NR |

Tdap : tetanos, diphteria, acellular pertussis vaccine

# DTaP(±HB)-IPV-Hib): pediatric combined diphtheria, tetanus, acellular pertussis, hepatitis B virus, inactivated poliovirus, and Haemophilus influenzae type b (DTaP(§HB)-IPV-Hib) vaccine (3 doses 1 month apart).

* statistically significant difference; § antibody titer considered to protect against tetanus: 0.06 HU/ml; $ seroconversion if antibody > 0.01 IU/ml

**Supplementary table 3: Summary of key published studies evaluating the immunogenicity diphteria vaccine in different adult populations**

| Study | Population | Age | Vaccine | Timepoint  After vaccine | Pre-vaccine Ab concentration | | Post-vaccine Ab concentration | | Fold response (definition) | Fold response (%) |  |
| --- | --- | --- | --- | --- | --- | --- | --- | --- | --- | --- | --- |
|  |  |  |  |  | ≥ 0.1 IU/mL | ≥1 IU/mL | ≥ 0.1 IU/mL | ≥1  IU/mL |  |  |  |
| Present study | 119 cirrhosis | 64  (58-70) | Tdap | 6 months | 46% | 8% | 66% | 11% | V1/V0≥ | 26% |  |
| **Healthy adults** | | | | | | | | | | |  |
| Halperin SA^47^ | 746 healthy adults | 33  (20-72) | Tdap | 1 month | 69% | 23% | 95.8%  (715/746) | 85%  (635/746) | V1/V0≥2 or V1/V0≥4 | 83.5%  (623/746) |  |
| Thierry-Carstensen B^38^ | 800 healthy adults | 26.1 (18-55) | 401Tdap  399 Td | 1 month | 56.7% | NR | 99% | NR | V1/V0≥2 or V1/V0≥4 | 96.9% |  |
| Van Damme P^39^ | 293 Adults ≥ 55 yr | 64.5±7.3 | Boostrix | 1 month | 45.2% | NR | 82.8% | NR | NR | NR |  |
| Weston WM^40^ | 887 adults ≥ 65 yr | 71.6±5.3 | Tdap | 1 month | 62% | 11.3% | 84.9% | 52% | NR | NR |  |
| **Patients with comorbidities** | | | | | | | | | | | |
| Caporuscio S^48^ | 38 rhumatoid arthritis | 62.4±11 | d | 1 month | 20% | NR | 68.6% | NR | NR | NR |  |
| Buhler S^41^ | 284 rheumatic diseases  253 healthy control | 52±14.4  45±15 | Td | 3 month | 48%  62%* | 5%  5% | 73%  84%* | 25%  25% | ≥2 at 1 month | 68% |  |
| Nunes MC^42^ | 91 pregnant HIV+  136 pregnant HIV- | 27.3±5.5  30.7±4.9 | Adacel |  | 7.9%  10.5% | 1.1%  1.5% | 65.1%  81.1%* | 18.6%  41.8%* | NR | NR |  |
| Conrad A^43^ | 106 Allo-HSCT | 47 (37.8-60) | DTaP(±HB)-IPV-Hib# | 6 months | 36% | NR | 95% | NR | NR | NR |  |
| Epaulard O^44^ | 104 Allo-HSCT | 58 (48-64) | DTaP(±HB)-IPV-Hib# | 1 month | 25.3% | NR | 100% | NR | NR | NR |  |

Tdap : tetanos, diphteria, acellular pertussis vaccine

# DTaP(±HB)-IPV-Hib): pediatric combined diphtheria, tetanus, acellular pertussis, hepatitis B virus, inactivated poliovirus, and Haemophilus influenzae type b (DTaP(§HB)-IPV-Hib) vaccine (3 doses 1 month apart). * statistically significant difference

**Supplementary Table 4: Summary of key published studies evaluating the immunogenicity of sequential vaccination with PCV13 followed by PPSV23 in different adult populations at high risk of pneumococcal infection**

|  | Population | Age | Immunological response at M4  V1/V0≥2 | Immunological protection at baseline | Immunological protection at M4 after vaccination |
| --- | --- | --- | --- | --- | --- |
| Present study | 119 Cirrhosis | 64  (58-70) | 48% | 39% | 80% |
| Bahuaud M^49^ | 24 Rheumatoid arthritis treated with MTX + anti-TNF | 62.5  (32-71) | 33% (8/24) | 38% | 63% |
| Garcia Garido HM ^50^ | Patients under immunosuppressive therapy  36 controls  165 immunosuppressed  47 under conventional immunomodulators  50 under biological immunomodulator  60 under combination therapy  21 Switched | 41  (26) | NR | 0  2.8%  4.3%  0  5%  0 | 85%  52%  53%  52%  46%  60% |
| van Aalst M^51^ | 141 patients with inflammatory bowel disease  37 non immunosuppressed IBD Controls  104 IBD under immunosuppression  35 under conventional immunomodulators  40 under biological immunomodulator  29 under combination therapy | 45  (29-56) | NR | 2.7%  1.9%  0  0  6.9% | 84%  50%  49%  58%  41% |
| Haggenburg S^52^ | 143 Chronic lymphocytic leukemia  38 treated  105 treatment naive | 66±9.2 | V1/V0≥4  10.5%  13.3%  2.6% |  | 10.5%  13.3%  2.6% |

Immunological response= 2-fold increase in IgG antibody for at least 5 of the 7 serotypes tested

Immunological protection= at least 5/7 antibodies with a concentration≥1.3 µg/ml

References

1. Chalasani N, Smallwood G, Halcomb J, Fried MW, Boyer TD. Is vaccination against hepatitis B infection indicated in patients waiting for or after orthotopic liver transplantation? *Liver Transpl Surg*. Mar 1998;4(2):128-32. doi:10.1002/lt.500040208

2. Villeneuve E, Vincelette J, Villeneuve JP. Ineffectiveness of hepatitis B vaccination in cirrhotic patients waiting for liver transplantation. *Can J Gastroenterol*. Jul-Aug 2000;14 Suppl B:59b-62b. doi:10.1155/2000/548206

3. Roni DA, Pathapati RM, Kumar AS, Nihal L, Sridhar K, Tumkur Rajashekar S. Safety and efficacy of hepatitis B vaccination in cirrhosis of liver. *Adv Virol*. 2013;2013:196704. doi:10.1155/2013/196704 [doi]

4. Kallinowski B, Benz C, Buchholz L, Stremmel W. Accelerated schedule of hepatitis B vaccination in liver transplant candidates. *Transplant Proc*. May 1998;30(3):797-9. doi:10.1016/s0041-1345(98)00053-0

5. Engler SH, Sauer PW, Golling M, et al. Immunogenicity of two accelerated hepatitis B vaccination protocols in liver transplant candidates. *Eur J Gastroenterol Hepatol*. Apr 2001;13(4):363-7. doi:10.1097/00042737-200104000-00010

6. Domínguez M, Bárcena R, García M, López-Sanroman A, Nuño J. Vaccination against hepatitis B virus in cirrhotic patients on liver transplant waiting list. *Liver Transpl*. Jul 2000;6(4):440-2. doi:10.1053/jlts.2000.8313

7. Macedo G, Maia JC, Gomes A, Teixeira A, Ribeiro T. Efficacy of a reinforced protocol of HBV vaccination in cirrhotic patients waiting for orthotopic liver transplantation. *Transplant Proc*. Dec 2000;32(8):2641. doi:10.1016/s0041-1345(00)01816-9

8. Rodriguez-Tajes S, Pocurull A, Lens S, et al. Efficacy of an accelerated double-dose hepatitis B vaccine regimen in patients with cirrhosis. *J Viral Hepat*. Jul 2021;28(7):1019-1024. doi:10.1111/jvh.13509

9. Bonazzi PR, Bacchella T, Freitas AC, et al. Double-dose hepatitis B vaccination in cirrhotic patients on a liver transplant waiting list. *Braz J Infect Dis*. Aug 2008;12(4):306-9. doi:10.1590/s1413-86702008000400009

10. Horlander JC, Boyle N, Manam R, et al. Vaccination against hepatitis B in patients with chronic liver disease awaiting liver transplantation. *Am J Med Sci*. Nov 1999;318(5):304-7. doi:10.1097/00000441-199911000-00004

11. Pascasio JM, Aoufi S, Gash A, et al. Response to a vaccination schedule with 4 doses of 40 microg against hepatitis B virus in cirrhotic patients evaluated for liver transplantation. *Transplant Proc*. Nov 2008;40(9):2943-5. doi:10.1016/j.transproceed.2008.09.029

12. Gutierrez Domingo I, Pascasio Acevedo JM, Alcalde Vargas A, et al. Response to vaccination against hepatitis B virus with a schedule of four 40-μg doses in cirrhotic patients evaluated for liver transplantation: factors associated with a response. *Transplant Proc*. Jul-Aug 2012;44(6):1499-501. doi:10.1016/j.transproceed.2012.05.071

13. Arslan M, Wiesner RH, Sievers C, Egan K, Zein NN. Double-dose accelerated hepatitis B vaccine in patients with end-stage liver disease. *Liver Transpl*. Apr 2001;7(4):314-20. doi:10.1053/jlts.2001.23069

14. De Maria N, Idilman R, Colantoni A, Van Thiel DH. Increased effective immunogenicity to high-dose and short-interval hepatitis B virus vaccination in individuals with chronic hepatitis without cirrhosis. *J Viral Hepat*. Sep 2001;8(5):372-6. doi:10.1046/j.1365-2893.2001.00301.x

15. Leroy V, Bourliere M, Durand M, et al. The antibody response to hepatitis B virus vaccination is negatively influenced by the hepatitis C virus viral load in patients with chronic hepatitis C: a case-control study. *Eur J Gastroenterol Hepatol*. May 2002;14(5):485-9. doi:10.1097/00042737-200205000-00004

16. Wiedmann M, Liebert UG, Oesen U, et al. Decreased immunogenicity of recombinant hepatitis B vaccine in chronic hepatitis C. *Hepatology*. Jan 2000;31(1):230-4. doi:10.1002/hep.510310134

17. Arbizu EA, Marugán RB, Grijalba JY, Serrano PL, Grande LG, Del Campo Terrón S. Intramuscular versus intradermal administration of anti-hepatitis B vaccine in non-cirrhotic hepatitis C patients. *Vaccine*. Jun 20 2003;21(21-22):2747-50. doi:10.1016/s0264-410x(03)00221-4

18. Horta D, Forne M, Agusti A, et al. Efficacy of Hepatitis B Virus Vaccines HBVaxpro40(c) and Fendrix(c) in Patients with Chronic Liver Disease in Clinical Practice. *Vaccines (Basel)*. Aug 16 2022;10(8)doi:10.3390/vaccines10081323

19. Medeiros RP, Terrault NA, Mazo DF, et al. Impaired anti-HBV vaccine response in non-cirrhotic chronic HCV is not overcome by double dose regimen: randomized control trial. *Ann Hepatol*. Mar-Apr 2023;28(2):100891. doi:10.1016/j.aohep.2022.100891

20. Amjad W, Alukal J, Zhang T, Maheshwari A, Thuluvath PJ. Two-Dose Hepatitis B Vaccine (Heplisav-B) Results in Better Seroconversion Than Three-Dose Vaccine (Engerix-B) in Chronic Liver Disease. *Dig Dis Sci*. Jun 2021;66(6):2101-2106. doi:10.1007/s10620-020-06437-6

21. Kwon JY, Daoud N, Ghoz H, Yataco ML, Farraye FA. Efficacy of a two-dose hepatitis B vaccination with a novel immunostimulatory sequence adjuvant (Heplisav-B) on patients with chronic liver disease: a retrospective study. *Transl Gastroenterol Hepatol*. 2023;8:8. doi:10.21037/tgh-22-12

22. Cruciani M, Mengoli C, Serpelloni G, et al. Serologic response to hepatitis B vaccine with high dose and increasing number of injections in HIV infected adult patients. *Vaccine*. Jan 1 2009;27(1):17-22. doi:10.1016/j.vaccine.2008.10.040

23. Launay O, van der Vliet D, Rosenberg AR, et al. Safety and immunogenicity of 4 intramuscular double doses and 4 intradermal low doses vs standard hepatitis B vaccine regimen in adults with HIV-1: a randomized controlled trial. *Jama*. Apr 13 2011;305(14):1432-40. doi:10.1001/jama.2011.351

24. Chaiklang K, Wipasa J, Chaiwarith R, Praparattanapan J, Supparatpinyo K. Comparison of immunogenicity and safety of four doses and four double doses vs. standard doses of hepatitis B vaccination in HIV-infected adults: a randomized, controlled trial. *PLoS One*. 2013;8(11):e80409. doi:10.1371/journal.pone.0080409

25. Rech-Medeiros AF, Marcon PDS, Tovo CDV, de Mattos AA. Evaluation of response to hepatitis B virus vaccine in adults with human immunodeficiency virus. *Ann Hepatol*. Sep-Oct 2019;18(5):725-729. doi:10.1016/j.aohep.2019.03.012

26. Fleming SJ, Moran DM, Cooksley WG, Faoagali JL. Poor response to a recombinant hepatitis B vaccine in dialysis patients. *J Infect*. May 1991;22(3):251-7. doi:10.1016/s0163-4453(05)80007-6

27. Bel'eed K, Wright M, Eadington D, Farr M, Sellars L. Vaccination against hepatitis B infection in patients with end stage renal disease. *Postgrad Med J*. Sep 2002;78(923):538-40. doi:10.1136/pmj.78.923.538

28. Janssen RS, Mangoo-Karim R, Pergola PE, et al. Immunogenicity and safety of an investigational hepatitis B vaccine with a toll-like receptor 9 agonist adjuvant (HBsAg-1018) compared with a licensed hepatitis B vaccine in patients with chronic kidney disease. *Vaccine*. Nov 4 2013;31(46):5306-13. doi:10.1016/j.vaccine.2013.05.067

29. Jackson S, Lentino J, Kopp J, et al. Immunogenicity of a two-dose investigational hepatitis B vaccine, HBsAg-1018, using a toll-like receptor 9 agonist adjuvant compared with a licensed hepatitis B vaccine in adults. *Vaccine*. Jan 29 2018;36(5):668-674. doi:10.1016/j.vaccine.2017.12.038

30. Van Der Meeren O, Peterson JT, Dionne M, et al. Prospective clinical trial of hepatitis B vaccination in adults with and without type-2 diabetes mellitus. *Hum Vaccin Immunother*. Aug 2 2016;12(8):2197-2203. doi:10.1080/21645515.2016.1164362

31. Han B, Liu W, Du J, et al. Immunogenicity and safety of hepatitis B vaccination in patients with type 2 diabetes in China: An open-label randomized controlled trial. *Vaccine*. Jun 8 2021;39(25):3365-3371. doi:10.1016/j.vaccine.2021.04.058

32. Belle A, Baumann C, Bigard MA, et al. Impact of immunosuppressive therapy on hepatitis B vaccination in inflammatory bowel diseases. *Eur J Gastroenterol Hepatol*. Aug 2015;27(8):877-81. doi:10.1097/meg.0000000000000370

33. Singh AK, Soni RK, Jearth V, et al. Clinical Trial: Immunogenicity of Double-Dose Versus Standard-Dose of Hepatitis B Virus Vaccine in Inflammatory Bowel Disease. *Aliment Pharmacol Ther*. Feb 2026;63(4):483-493. doi:10.1111/apt.70470

34. Etzion O, Novack V, Perl Y, et al. Sci-B-VacTM Vs ENGERIX-B Vaccines for Hepatitis B Virus in Patients with Inflammatory Bowel Diseases: A Randomised Controlled Trial. *J Crohns Colitis*. Aug 2016;10(8):905-12. doi:10.1093/ecco-jcc/jjw046

35. Heyward WL, Kyle M, Blumenau J, et al. Immunogenicity and safety of an investigational hepatitis B vaccine with a Toll-like receptor 9 agonist adjuvant (HBsAg-1018) compared to a licensed hepatitis B vaccine in healthy adults 40-70 years of age. *Vaccine*. Nov 4 2013;31(46):5300-5. doi:10.1016/j.vaccine.2013.05.068

36. Sablan BP, Kim DJ, Barzaga NG, et al. Demonstration of safety and enhanced seroprotection against hepatitis B with investigational HBsAg-1018 ISS vaccine compared to a licensed hepatitis B vaccine. *Vaccine*. Mar 30 2012;30(16):2689-96. doi:10.1016/j.vaccine.2012.02.001

37. Vesikari T, Langley JM, Segall N, et al. Immunogenicity and safety of a tri-antigenic versus a mono-antigenic hepatitis B vaccine in adults (PROTECT): a randomised, double-blind, phase 3 trial. *Lancet Infect Dis*. Sep 2021;21(9):1271-1281. doi:10.1016/s1473-3099(20)30780-5

38. Thierry-Carstensen B, Jordan K, Uhlving HH, et al. A randomised, double-blind, non-inferiority clinical trial on the safety and immunogenicity of a tetanus, diphtheria and monocomponent acellular pertussis (TdaP) vaccine in comparison to a tetanus and diphtheria (Td) vaccine when given as booster vaccinations to healthy adults. *Vaccine*. Aug 10 2012;30(37):5464-71. doi:10.1016/j.vaccine.2012.06.073

39. Van Damme P, McIntyre P, Grimprel E, et al. Immunogenicity of the reduced-antigen-content dTpa vaccine (Boostrix(®)) in adults 55 years of age and over: a sub-analysis of four trials. *Vaccine*. Aug 11 2011;29(35):5932-9. doi:10.1016/j.vaccine.2011.06.049

40. Weston WM, Friedland LR, Wu X, Howe B. Vaccination of adults 65 years of age and older with tetanus toxoid, reduced diphtheria toxoid and acellular pertussis vaccine (Boostrix(®)): results of two randomized trials. *Vaccine*. Feb 21 2012;30(9):1721-8. doi:10.1016/j.vaccine.2011.12.055

41. Bühler S, Jaeger VK, Adler S, et al. Safety and immunogenicity of tetanus/diphtheria vaccination in patients with rheumatic diseases-a prospective multi-centre cohort study. *Rheumatology (Oxford)*. Sep 1 2019;58(9):1585-1596. doi:5382022 [pii]

10.1093/rheumatology/kez045 [doi]

42. Nunes MC, Tamblyn A, Jose L, et al. Immunogenicity of tetanus, diphtheria and acellular pertussis vaccination among pregnant women living with and without HIV. *Aids*. Dec 1 2023;37(15):2305-2310. doi:10.1097/qad.0000000000003731

43. Conrad A, Perry M, Langlois ME, et al. Efficacy and Safety of Revaccination against Tetanus, Diphtheria, Haemophilus influenzae Type b and Hepatitis B Virus in a Prospective Cohort of Adult Recipients of Allogeneic Hematopoietic Stem Cell Transplantation. *Biol Blood Marrow Transplant*. Sep 2020;26(9):1729-1737. doi:10.1016/j.bbmt.2020.05.006

44. Epaulard O, Carré M, Hermet E, et al. Antibody response to tetanus, diphtheria, poliomyelitis, hepatitis B, and H. influenzae b vaccines in allogeneic hematopoietic stem cell transplant adult recipients: A multicenter trial. *PLoS One*. 2025;20(10):e0335224. doi:10.1371/journal.pone.0335224

45. Guerin A, Buisson Y, Nutini MT, Saliou P, London G, Marchais S. Response to vaccination against tetanus in chronic haemodialysed patients. *Nephrol Dial Transplant*. 1992;7(4):323-6. doi:10.1093/oxfordjournals.ndt.a092136

46. Girndt M, Pietsch M, Köhler H. Tetanus immunization and its association to hepatitis B vaccination in patients with chronic renal failure. *Am J Kidney Dis*. Sep 1995;26(3):454-60. doi:10.1016/0272-6386(95)90491-3

47. Halperin SA, Scheifele D, De Serres G, et al. Immune responses in adults to revaccination with a tetanus toxoid, reduced diphtheria toxoid, and acellular pertussis vaccine 10 years after a previous dose. *Vaccine*. Jan 20 2012;30(5):974-82. doi:10.1016/j.vaccine.2011.11.035

48. Caporuscio S, Ieraci R, Valesini G, et al. Anti-polysaccharide and anti-diphtheria protective antibodies after 13-valent pneumococcal conjugate vaccination in rheumatoid arthritis patients under immunosuppressive therapy. *Clin Immunol*. Oct 2018;195:18-27. doi:10.1016/j.clim.2018.07.010

49. Bahuaud M, Beaudouin-Bazire C, Husson M, et al. Immunogenicity and persistence of a prime-boost re-vaccination strategy for pneumococcal vaccines in patients with rheumatoid arthritis. *Hum Vaccin Immunother*. Jun 3 2018;14(6):1464-1470. doi:10.1080/21645515.2018.1438091

50. Garcia Garrido HM, Vollaard A, D'Haens GR, et al. Immunogenicity of the 13-Valent Pneumococcal Conjugate Vaccine (PCV13) Followed by the 23-Valent Pneumococcal Polysaccharide Vaccine (PPSV23) in Adults with and without Immunosuppressive Therapy. *Vaccines (Basel)*. May 17 2022;10(5)doi:10.3390/vaccines10050795

51. van Aalst M, Garcia Garrido HM, van der Leun J, et al. Immunogenicity of the Currently Recommended Pneumococcal Vaccination Schedule in Patients With Inflammatory Bowel Disease. *Clin Infect Dis*. Feb 3 2020;70(4):595-604. doi:10.1093/cid/ciz226

52. Haggenburg S, Garcia Garrido HM, Kant IMJ, et al. Immunogenicity of the 13-Valent Pneumococcal Conjugated Vaccine Followed by the 23-Valent Polysaccharide Vaccine in Chronic Lymphocytic Leukemia. *Vaccines (Basel)*. Jul 4 2023;11(7)doi:10.3390/vaccines11071201
